# Frequency and outcomes of benign breast biopsies in trans women: A nationwide cohort study

**DOI:** 10.1016/j.breast.2021.03.007

**Published:** 2021-03-25

**Authors:** Christel JM. de Blok, Benthe AM. Dijkman, Chantal M. Wiepjes, Inge RHM. Konings, Koen MA. Dreijerink, Ellis Barbé, Martin den Heijer

**Affiliations:** a Center of Expertise on Gender Dysphoria, Amsterdam UMC, VU University Medical Center, De Boelelaan 1117, 1081 HV, Amsterdam, the Netherlands; b Department of Endocrinology, Amsterdam UMC, VU University Medical Center, De Boelelaan 1117, 1081 HV, Amsterdam, the Netherlands; c Department of Oncology, Amsterdam UMC, VU University Medical Center, De Boelelaan 1117, 1081 HV, Amsterdam, the Netherlands; d Department of Pathology, Amsterdam UMC, VU University Medical Center, De Boelelaan 1117, 1081 HV, Amsterdam, the Netherlands

**Keywords:** Transgender, Trans women, Breast lesions, Pathology, Gender-affirming hormone treatment, Estrogen

## Abstract

No literature is available on the benign versus malignant breast lesion ratio in trans women (male sex assigned at birth, female gender identity). As hormone treatment in trans women results in breast tissue histologically comparable with cis (non-trans) women, breast pathology may be expected. Previously, an increased breast cancer risk compared with cis men have been observed. We aimed to investigate the frequency and outcomes of breast biopsies in trans women. Therefore, we retrospectively examined the medical files of 2616 trans women. To gain data on breast lesions, we linked our cohort to a national pathology database. In this study we found that 126 people (5%) had one or more breast biopsies (n = 139). Of these, 21 trans women had a breast biopsy before the start of hormone treatment, and 53 after the start of hormone treatment. Breast biopsies were performed predominantly because of abnormalities during physical examination (37%, n = 51/139 biopsies), or because of capsular formation or contraction (28%, n = 16/57 biopsies) in trans women with breast implants. The most common breast lesions after the start of hormone treatment were fibroadenomas (n = 20), breast cancer (n = 6), fibrosis (n = 5), cysts (n = 4), and infections (n = 4). The benign versus malignant breast biopsy ratio was 88:12, which is comparable to the ratio in cis women (90:10). This study shows breast lesions in a limited number of trans women. Since the indications and outcomes of biopsies in trans women were similar to those in cis women, it seems reasonable to follow breast care guidelines as developed for cis women.

## 1 Introduction

Gender dysphoria is defined as an incongruence between one’s sex assigned at birth and one’s gender identity [1]. To induce physical changes in line with the gender identity, trans people may seek gender-affirming treatment which may include hormone treatment. In trans women (male sex assigned at birth, female gender identity), hormone treatment usually consists of a combination of antiandrogens and estrogens to induce feminization, including breast development [2,3].

Mammary tissue development is similar in cis boys (alignment of sex assigned at birth and gender identity) and cis girls until puberty. During typical female puberty, breast development occurs influenced by female sex hormones [[4], [5], [6]]. Besides (active) glandular tissue with ducts and lobules, the mature cis female breast consists of skin, stromal elements, and fat [7]. Due to exposure to testosterone during typical male puberty, no further mammary tissue development occurs in cis boys [4,8]. Eventually, the mature cis male breast consists of skin, primitive ducts, stromal elements, and fat tissue [7]. Because of the different breast structure between cis men and cis women, lobular pathology, such as fibroadenomas and cysts, is not expected in cis men. However, some reports of fibroadenomas in cis men have been published [9]. Benign breast lesions that are more often observed in cis men are gynecomastia, dermal cysts, lipomas and angiolipomas, mastitis and abscesses, granular cell tumors, and pseudoangiomatous stromal hyperplasia (PASH) [7]. Influenced by exogenous female sex hormones, the male breast can further develop into a histological female breast as seen in trans women receiving gender-affirming hormone treatment [8].

As hormone-induced breast development in trans women results in a histological female breast with ducts and lobules, lobular pathology in trans women may be expected [10]. Indeed, cases of lobular pathology in trans women including fibroadenomas have been published [[11], [12], [13]]. However, the incidence of these lesions in trans women is unknown. Moreover, the ratio of benign versus malignant lesions in this group, which is 90:10 in cis women [14], is also unknown. Therefore, the aim of this nationwide study was to describe the frequency and outcomes of breast biopsies in a well-defined, large cohort of trans women.

## 2 Materials & methods

### 2.1 Study population

Trans women who started hormone treatment between 1991 and 2018 at the Center of Expertise on Gender Dysphoria at the Amsterdam UMC, the Netherlands were eligible for participation [15]. People were excluded from analyses if they were younger than 18 years of age at the time of this study, if they used testosterone, or if the start date of hormone treatment was unknown. The subject selection only partially overlaps between the current and our previously published study on breast cancer in trans people [16]. For the current study, only trans women who started hormone treatment after 1990 were included, whereas in our previous study people who started hormone treatment between 1972 and 2018 were included. The subject selection in these two studies differed because we used the database of the Nationwide Network and Registry of Histopathology and Cytopathology in the Netherlands (PALGA) which only has nationwide coverage since 1991. Moreover, medical history in the medical files always include past history of cancer, but not necessarily on benign breast lesions. Therefore, we only included people who started hormone treatment since 1991 to make sure we did not miss any benign breast lesion case.

Most trans women were treated with a combination of antiandrogens and estrogens. Antiandrogen therapy usually consisted of cyproterone acetate (a progestogenic antiandrogen, 10–100 mg daily) or spironolactone (100–200 mg daily), and was often ceased after orchiectomy. Estrogen was prescribed as ethinylestradiol (25–100 mcg daily), conjugated estrogens (0.625–1.25 mg daily), estradiol patches (50–150 mcg/24 h twice weekly), estradiol implants (20 mg every 3–6 months), estradiol injections (10–100 mg every 2–4 weeks), estradiol valerate (2–6 mg daily), or estradiol gel (0.75–3 mg daily). In recent years, mainly estradiol valerate, estradiol patches, or estradiol gel were used. Trans women who started with hormone treatment under the age of 18 years often started with gonadotrophin-releasing hormone agonists only before addition of estrogen.

This study was reviewed by the Ethical Review Board of the Amsterdam UMC, VU University Medical Center Amsterdam. It was determined that the Medical Research Involving Human Subjects Act (WMO) does not apply to this study, and necessity for informed consent was waived. All data were processed anonymously.

### 2.2 Data collection

Of all eligible people, data on age at start of hormone treatment, gender-affirming surgery, mean body mass index (BMI) during follow-up, and medical history were collected. Data on breast pathology diagnoses were retrieved from the Nationwide Network and Registry of Histopathology and Cytopathology in the Netherlands (PALGA) after linkage of the study cohort to their database [17]. Data will not be shared at an individual level to guarantee the anonymity of the people in the database.

### 2.3 Statistical analysis

Baseline data are presented as mean with standard deviation (SD) for normally distributed data. Non-normally distributed data are presented as median with inter-quartile range (IQR). Breast lesions are presented as number of diagnoses with percentage. Of the people who started hormone treatment before their treatment at our clinic, the first known start date of hormone treatment was used to calculate treatment duration. Treatment duration was calculated from the start date of hormone treatment to either first breast lesion, end of study period (31/12/2018), or date of death. As trans women may have more than one breast lesions, the number of breast lesions may exceed the number of trans women with breast lesions.

All analyses were performed using STATA statistical Software, version 15.1 (Statacorp, College Station, Texas, USA).

## 3 Results

In total, 2616 trans women were included in this study of whom 126 (5%) had diagnosed breast lesions. The median age at start of hormone treatment was 30 years (IQR 23 to 41). During follow-up, 58% of the included trans women underwent an orchiectomy. No trans women underwent orchiectomy before entering the study. The median follow-up time was 9 years (IQR 3 to 18) which resulted in a total follow-up time of the study cohort of 30,311 years. The characteristics of the study cohort are summarized in Table 1.

**Table 1:** Baseline characteristics.

	N = 2616
Age at start of HT, y	30 (23–41)
BMI, kg/m^2^	24.7 (21.2–26.5)
Orchiectomy, n (%) yes	1528 (58)
Median follow-up time, y	9 (3–18)
Total follow-up time, y	30,311

Data are presented as median with inter-quartile range, percentage or number of years.

Abbreviations: HT = hormone treatment; BMI = body mass index.

In the 126 trans women with breast lesions, 139 diagnoses were observed. Twenty-one trans women had a breast lesion before the start of hormone treatment, 53 after the start of hormone treatment, and in 56 trans women breast lesions were associated with the presence of silicone breast implants. Some trans women have been diagnosed with multiple breast lesions during follow-up.

In trans women with breast lesions after the start of hormone treatment, biopsies were performed after median 20 years (IQR 16 to 22) of hormone treatment. As shown in Table 2, breast biopsies were performed mostly because of abnormalities observed during physical examination or abnormalities at imaging studies. The most commonly observed breast lesions in this group were fibroadenomas, invasive breast cancer, fibrosis, cysts, and infections. No cases of gynecomastia could be associated to medication use based on the available data. The observed breast cancer cases have been described earlier [16], and therefore will not be further discussed in this paper. The number of breast cancer cases differs from our previous study, because the selection of participants overlaps only partially between the two studies (see Methods section). The benign versus malignant lesion ratio in trans women with observed breast lesions after the start of hormone treatment was 88:12.

**Table 2:** Breast lesions (n = 60) in 53 trans women after the start of hormone treatment^^^.

Reason for breast biopsies	N (%)
Physical examination abnormalities	27 (45)
Imaging abnormalities	25 (42)
Preventive surgery	3 (5)
Cosmetic ∗	1 (2)
Unknown	4 (7)
**Diagnoses**	
Fibroadenoma	20 (33)
Breast cancer	6 (10)
Fibrosis	5 (8)
Cyst	4 (7)
No abnormalities	4 (7)
Infection	4 (7)
Mastopathy	3 (5)
Hyperplasia	3 (5)
Gynecomastia	2 (3)
DCIS	1 (2)
Other∗∗	8 (13)

Data are presented as number (%). ^^^ Some trans women had multiple diagnoses ∗ breast tissue lift ∗∗ Including adenosis (n = 2), microcalcification (n = 1), duct ectasias (n = 1), cystic enlarged ducts (n = 1), lymph node tissue (n = 1), papilloma (n = 1), cylindrical cell changes (n = 1) Abbreviations: DCIS = ductal carcinoma in situ.

In trans women in whom breast lesions were associated with the presence of silicone breast implants, breast biopsies were mostly performed because of capsular formation or contraction (28%, n = 16/57 biopsies, Table 3). Other reasons for breast biopsy included abnormalities during physical examination (19%, n = 11/57 biopsies), surgery associated with the recall of the Poly Implants Prostheses (PIP) implants (18%, n = 10/57 biopsies), implant leakage (12%, n = 7/57 biopsies), implant replacement (9%, n = 5/57 biopsies), and injection with silicone oil (4%, n = 2/57 biopsies). In most cases, a histological reaction to silicone was observed (72%, n = 41/57 biopsies). Other common diagnoses included visible silicone particles (5%, n = 3/57 biopsies), sclerosis/calcification (5%, n = 3/57 biopsies), and silicone granulomas (4%, n = 2/57 biopsies).

**Table 3:** Breast lesions (n = 57) of 56 trans women with silicone breast implants^^^.

Reason for breast biopsies	N (%)
Capsular formation/contraction	16 (28)
Abnormalities in physical examination	11 (19)
PIP (recall)	10 (18)
Implant leakage	7 (12)
Implant replacement	5 (9)
Injection with silicone oil	2 (4)
Unknown	6 (11)
**Diagnoses**	
Reaction to silicone	41 (72)
No abnormalities	5 (9)
Visible silicone particles	3 (5)
Sclerosis/calcification	3 (5)
Silicone granuloma	2 (4)
Other ∗	3 (5)

Data are presented as number (%). ^^^ One trans woman had two diagnoses.∗ Including no diagnosis (no cells for diagnosis, n = 1), silicone oil (n = 1), and seroma (n = 1) Abbreviations: PIP; Poly Implant Prostheses.

Gynecomastia (82%, n = 18/22 biopsies) was the most common breast lesion in trans women with a breast lesion before the start or hormone treatment (Table 4). Other lesions included fibroadenoma (9%, n = 2/22 biopsies), cysts (5%, n = 1/22 biopsies), and fibrosis (5%, n = 1/22 biopsies). Four cases of gynecomastia could be associated to medication use based on the available data.

**Table 4:** Breast lesions (n = 22) in 21 trans women before the start of hormone treatment^^^.

Reason for breast biopsies	N (%)
Palpable abnormalities	11 (50)
Visible abnormalities	2 (9)
Unknown	9 (41)
**Diagnoses**	
Gynecomastia	18 (82)
Fibroadenoma	2 (9)
Cyst	1 (5)
Fibrosis	1 (5)

Data are presented as number (%). ^^^ One trans woman had a lesion in both breasts.

## 4 Discussion

### 4.1 Principle findings

This study observed a benign versus malignant breast lesion ratio of 88:12 in trans women receiving hormone treatment. Breast biopsies were mostly performed because of a palpable mass, abnormalities at imaging, or because of capsular formation or contraction in trans women with silicone breast implants. Before the start of hormone treatment, the most common breast lesion was gynecomastia whereas after the start of hormone treatment fibroadenomas, breast cancer, fibrosis, cysts, and infections were most commonly diagnosed. In trans women with silicone breast implants, only benign reactions to silicone were observed.

### 4.2 Comparison with other studies

The benign versus malignant breast lesion ratio in trans women who underwent breast biopsies after the start of hormone treatment was 88:12 in this cohort. It is reassuring that this ratio is comparable to the ratio in cis women (90:10) [14]. Therefore, we advise to follow the guidelines for breast care in cis women for trans women as well, without any extra precautions or tests for breast lesions or breast cancer.

Kanhai et al. [18] previously described that breast histology in trans women using hormone treatment was comparable with histology in cis women. Therefore, we were not surprised to observe several cases of fibroadenomas and cysts in our study. With an overall prevalence of 0.9% (n = 24/2616) in this cohort of trans women, the prevalence of benign lobular breast lesions was lower than reported in cis women populations [19,20]. A possible explanation for this difference might be that the trans women in our cohort have been exposed to estrogens for a shorter period of their lives than cis women. Furthermore, because of hormone fluctuations (e.g. during pregnancy) fibroadenomas can grow [20]. The usually fixed dose hormone treatment in trans women may therefore also contribute to a lower observed prevalence of fibroadenomas in trans women compared with cis women. Trans women from 50 years of age who are registered as female will receive an invite to participate in the breast cancer screening program in the Netherlands automatically. Maybe, trans women participate less in this screening program than cis women. This may contribute to the low number of observed (benign) breast lesions in trans women compared with cis women.

The overall prevalence of gynecomastia in this study was 0.8% (n = 20/2616), whereas the prevalence of clinical diagnoses of gynecomastia in adult cis men populations (age between 50 and 80 years) ranges between 24% and 65% [21,22]. Despite the low overall prevalence, the prevalence of gynecomastia cases associated with the use of medication in this study was comparable with the prevalence of medication use associated cases in cis men (20% and 10–25%, respectively) [21]. A possible reason for the difference in gynecomastia prevalence between trans women and cis men, is that trans women before the start of hormone treatment do not present with complaints of gynecomastia, because breast enlargement is in line with their gender identity. On the other hand, 18 breast biopsies for gynecomastia is remarkable. Maybe trans women are more focused on their breast and therefore present themselves quicker with changes in their breasts which leads to biopsy. Interestingly, three fibroadenomas and two cysts were observed in trans women before the start of hormone treatment. Although lobular pathology rarely occurs in the male breast [7], it has already been described in literature [23].

Based on previous research, it is estimated that 70–80% of trans women who received hormone treatment also underwent breast augmentation surgery [[24], [25], [26], [27]]. Therefore, it is estimated that 1831 to 2093 trans women have breast implants in this study cohort. Of the (estimated) number of subjects with breast implants, only 56 (3%) trans women underwent a breast biopsy. With this estimation, breast biopsies were performed in 1:33 to 1:37 trans women with silicone breast implants. Breast biopsies were mostly performed because of capsular formation or contraction (28%), a known and common complication of silicone breast implants [28]. However, this is probably an underestimation as not all capsular formation or contraction will lead to surgery and consequently the opportunity for breast biopsies. Moreover, it is very likely that not all removed implant capsules have been sent to the pathologist. This may also explain the relative low occurrence of capsular contraction observed in this study [28]. Importantly, we only observed benign breast lesions in trans women with breast implants who underwent breast biopsies and did not observe any case of breast implant associated anaplastic large cell lymphoma (BIA-ALCL) [29].

### 4.3 Strengths and limitations

This large cohort study allows for an accurate estimation of the prevalence of breast lesions in trans women. Furthermore, this study included people with a wide range of ages and had a long follow-up time. However, this study has some limitations as well. First, as most of the breast biopsies were performed in other hospitals and the clinical data field in the PALGA database was not always filled out, the reasons why breast biopsies were performed was not always known. Second, because of the retrospective design of this study, data on for instance other medication use may be incomplete or missing. Therefore, it is possible that we underestimate the prevalence of gynecomastia associated with medication use in this study.

## 5 Conclusions

This study shows breast lesions in a small number of trans women receiving hormone treatment. Whereas gynecomastia was the most observed diagnosis in trans women before the start of hormone treatment, pathology such as fibroadenomas, breast cancer, fibrosis, cysts, and infections were most commonly diagnosed after the start of hormone treatment. The benign versus malignant breast lesion ratio was 88:12, which is comparable to the ratio observed in cis women. We advise to follow the guidelines for breast care and breast cancer screening in cis women for trans women as well.

## Ethical approval

This study was reviewed by the Ethical Review Board of the Amsterdam UMC, VU University Medical Center Amsterdam. It was determined that the Medical Research Involving Human Subjects Act (WMO) does not apply to this study, and necessity for informed consent was waived.

## Funding

No funding was received for this study.

## Declaration of competing interest

The authors report no conflict of interest.
